# Clinical impact of the metabolic phenotype of prostate cancer: role of monocarboxylate transporters (MCTs)

**DOI:** 10.1186/1753-6561-7-S2-P53

**Published:** 2013-04-04

**Authors:** Nelma Pértega-Gomes, José R Vízcaíno, Vera Miranda-Gonçalves, Carlos Lopes, Fátima Baltazar

**Affiliations:** 1 Life and Health Sciences Research Institute (ICVS), School of Health Sciences, University of Minho, Braga, Portugal; 2 ICVS/3B’s - PT Government Associate Laboratory, Braga/Guimarães, Portugal; 3 Department of Pathology, Centro Hospitalar do Porto, Portugal

## Background

Monocarboxylate transporters (MCTs) are transmembrane proteins that facilitate the transport of important monocarboxylates such as lactate across the cell membrane. Thus, these transporters play a central role in tumour metabolism and, as a result, are attractive targets in cancer therapy, which are now starting to be explored in the clinical context.

## Materials and methods

Expression of key metabolic markers was assessed by immunohistochemistry in 480 prostate samples and prostate cell lines. The levels of glycolytic metabolism were assessed using commercial colorimetric assays. The effect of the MCT inhibitor and thioridazine was evaluated on cell viability by using the Sulforhodamine B assay. Finally, ultra-structural studies were performed by classical electron microscopy transmission techniques.

## Results

Firstly, we observed an overexpression of proteins involved in oxidative phosphorylation and lipidic β-oxidation in localized prostate cancer, which expression was already evident in precursor lesions. Importantly, only proteins involved in glycolytic metabolism were associated with poor prognosis and the same proteins, despite expressed at low levels in localized tumour, were highly expressed in the metastatic samples. Secondly, prostate cancer cell lines showed important differences at metabolic and ultrastructural levels. The less aggressive LNCaP cells exhibited a more oxidative phenotype whereas the highly aggressive and metastatic cell lines PC3 and DU145 were more glycolytic. Finally, the distant metastatic prostate cancer cell lines were more sensitive to CHC (MCT inhibitor) than LNCaP cells.

## Conclusions

These studies demonstrate differences in the metabolism of prostate cancer cells, which could be relevant on the development of new diagnostic, prognostic and therapeutic strategies involving metabolic targets.

## Financial support

Fundação para a Ciência e Tecnologia (FCT).

